# Hormesis effects of phosphorus on the viability of *Chlorella regularis* cells under nitrogen limitation

**DOI:** 10.1186/s13068-019-1458-z

**Published:** 2019-05-13

**Authors:** Liang Fu, Qingcheng Li, Ge Yan, Dandan Zhou, John C. Crittenden

**Affiliations:** 1 Engineering Lab for Water Pollution Control and Resources Recovery, School of Environment, Northeast Normal University, Changchun, 130117 People’s Republic of China; 2 Brook Byers Institute for Sustainable Systems, and School of Civil & Environmental Engineering, Georgia Institute of Technology, Atlanta, GA 30332 USA

**Keywords:** Microalgae, Phosphorus, Hormesis, Nitrogen limitation, Toxic

## Abstract

**Background:**

Phosphorus (P) is an essential element of microalgae, which is either required for anabolism or for energy metabolism. When employing a nitrogen limitation strategy to trigger microalgal intracellular lipid accumulation, P supplementation was always simultaneously applied to compensate for the accompanied growth inhibition.

**Results:**

This study identified that P exerts hormesis effects on microalgae. Slight excess of P (≤ 45 mg L^−1^) under nitrogen limitation condition stimulated the cell growth of *Chlorella regularis* and achieved a 10.2% biomass production increase. This also improved mitochondrial activity by 25.0% compared to control (P = 5.4 mg L^−1^). The lipid productivity reached 354.38 mg (L d)^−1^, which increased by 39.3% compared to control. Such an improvement was caused by the intracellularly stored polyphosphate energy pool. However, large excess of P (250 mg L^−1^) inhibited the cell growth by 38.8% and mitochondrial activity decreased by 71.3%. *C. regularis* cells showed obvious poisoning status, such as enlarged size, plasmolysis, deformation of cell walls, and disorganization of organelles. This is probably because the over-accumulated P protonated the amide-N and disrupted membrane permeability.

**Conclusions:**

These results provide new insight into the roles of P in microalgae lipid production: P does not always play a positive role under nitrogen limitation conditions.

**Electronic supplementary material:**

The online version of this article (10.1186/s13068-019-1458-z) contains supplementary material, which is available to authorized users.

## Background

Microalgae are a promising feedstock for biodiesel [1, 2]. In their studies on microalgae bioenergy technologies in the past 40 years, researchers have endeavored to explore strategies for enhancing biomass production and lipid accumulation [3, 4], which taken together, determines microalgal lipid production. Nitrogen limitation is the most popular approach to increase the microalgal lipid content, because it is able to up-regulate the key enzymes on the lipid biosynthesis pathways: malic enzymes become more activated for catalyzing pyruvate synthesis and NADPH (reduced nicotinamide adenine dinucleotide phosphate) formation and both acetyl-CoA and ATP (adenosine triphosphate)-citrate lyase are stimulated for catalyzing acetyl-CoA production [5, 6]. The lipid content could be enhanced by 10–50% when nitrogen was limited [4, 7], and even up to two- to three-fold during nitrogen starvation [8, 9].

Even though nitrogen deprivation/limitation promoted intracellular lipid accumulation, it also decreased biomass production by as much as 80%, and caused the failure of final lipid production improvement [10, 11]. Nitrogen deprivation/limitation could weaken proteins synthesis, and thus induce the depletion of ADP (adenosine diphosphate) and NADP (nicotinamide adenine dinucleotide phosphate) and the dysfunction of cell synthesis [12, 13]. Phosphorus (P) supplementation offered an effective strategy to resolve these problems and has thus been employed to enhance microalgae biomass production under nitrogen limitation [14, 15]. Combining nitrogen limitation with P supplementation could contribute 10–100% increase on the microalgae biomass production, accompanied by 15–50% improvement of lipid accumulation, and finally achieved 50–160% enhancement of lipid productivity [4, 15, 16]. In a P-repletion condition, P was stored as intracellular polyphosphate (poly-P) by microalgae both in autotrophic and heterotrophic growth [17, 18]. Poly-P, with phosphoanhydride bonds, was rich in energy (e.g., ATP), which could be utilized for biosynthesis when cells were nitrogen deprived [14]. In addition, poly-P can also be metabolized to form DNA (deoxyribonucleic acid), RNA (ribonucleic acid), or intermediate products in microalgae [5]. Thus, replete phosphorus has traditionally been suggested to benefit microalgae growth and lipid accumulation [4, 11].

Excess P is advantageous for microalgal lipid production; however, it also increases the cost of cultivation. Agricultural and industrial wastewater are promising P sources. Piggery wastewater, dairy wastewater, brewery wastewater, food processing wastewater, and rubber mill wastewater, are all rich in P [19–22]. The P concentrations in these industrial wastewaters range widely from 3 to 330 mg L^−1^. However, previous studies on P supplementation improved microalgal cultivation, which was limited by the slight excess of P (≤ 45 mg L^−1^). The question remains whether large excess of P can continuously increase the lipid production of microalgae. The answer may be negative. Whether an environmental agent is beneficial or toxic depends on its dosage, which is referred to as hormesis. Hormesis is a biphasic dose–response to an environmental agent, i.e., a low dose stimulation has a beneficial effect and a high dose has inhibitory or toxic effects [23]. Based on this, slight excess of P (≤ 45 mg L^−1^) could stimulate cell growth [4]; however, the effects of large P excess still remain unknown. The results of this study provide new insight for the application of microalgae in P-bearing wastewater treatment.

In this study, the roles of P on *Chlorella regularis* (*C. regularis*, model strain) were investigated in a wide concentration range (5.4–250 mg-P L^−1^) under nitrogen limiting conditions. The effects of P concentration on cell growth (which were determined via cell density changes and substrates consumption), and intracellular lipid accumulation (which was related to the intracellular contents) were studied. In addition, mitochondrial activity assay, ultrastructure morphology of cells, intracellular-P storage forms, and chemical bond analyses were conducted, to identify the potential mechanism underlying P toxicity. Interestingly, hormesis effects of P on microalgae cells were confirmed, which specified the toxic of high P concentration on microalgae for the first time.

## Methods

### Microalgae strain, cultivation, and protocols

As a typical microalga, *C. regularis* var. *minima* (FACHB-729) was used in this study, which was purchased from the Freshwater Algae Culture Collection of the Institute of Hydrobiology in Wuhan, China. Prior to the experiments, *C. regularis* was purified via the streaking plating method and preserved on agar jelly at 4 °C. Before the experiment, *C. regularis* was activated using classic BG11 medium under photoautotrophic condition [24]. As an inoculum of the experiment, the activated *C. regularis* cells were collected at the logarithmic phase (20 days of phototrophic cultivation) via centrifuging (6000×*g* for 5 min at 4 °C), and then washed and re-suspended three times with 0.9% sterile physiological saline. After the above procedures, *C. regularis* was inoculated into the modified BG11 media, which resulted in an initial cell density of 1.5 × 10^7^ cell mL^−1^ for further heterotrophic at 28 °C, 160 rpm, in the dark (ZWY-240, Zhicheng Shanghai, China). During the experiments, all sampling operations were accomplished in a clean bench (SW-CJ-1FD, Airtech, Suzhou, China).

Real wastewater with high P concentration also has high levels of organics [21, 25]. Accordingly, glucose was selected to simulate organic carbon for microalgae heterotrophic cultivation [11, 26]. In this study, 10 g L^−1^ glucose (sterilized using sterile 0.22 mm filter) was mixed to the BG11 mediums to create heterotrophic cultivation. Nitrogen limitation was controlled by adding 300 mg L^−1^ NaNO_3_. The form of nitrogen was identical to the BG11 medium; however, its content was only 20% of the classic BG11 [4]; therefore, this condition was named nitrogen limitation (N_lim_). Furthermore, a series of phosphorus (PO_4_^3−^–P) levels were proposed to elaborate the effects of P concentration (mg L^−1^), 5.4, 25, 45, 150, and 250 mg L^−1^. The 5.4 mg-P L^−1^ was equal to the P concentration of the classic BG11 medium; therefore, it was used as control protocol (N_lim_P5.4). The others were called N_lim_P25, N_lim_P45, N_lim_P150, and N_lim_P250 in the following. Additionally, MgSO_4_, NaCO_3_, CaCl_2_, citric acid, ammonium ferric citrate, Na_2_EDTA, H_3_BO_3_, MnCl_2_, ZnSO_4_, CuSO_4_, CoCl_2_, and NaMoO_4_ were also supplemented as nutrients and trace elements according to a previous study [11]. The medium was autoclaved at 121 °C for 30 min, and the final pH was about 7.1 adjusted with sterile HCl and NaOH solution and no precipitation appeared in the medium.

### Growth profiles and nutrient consumptions

**Fig. 1:**
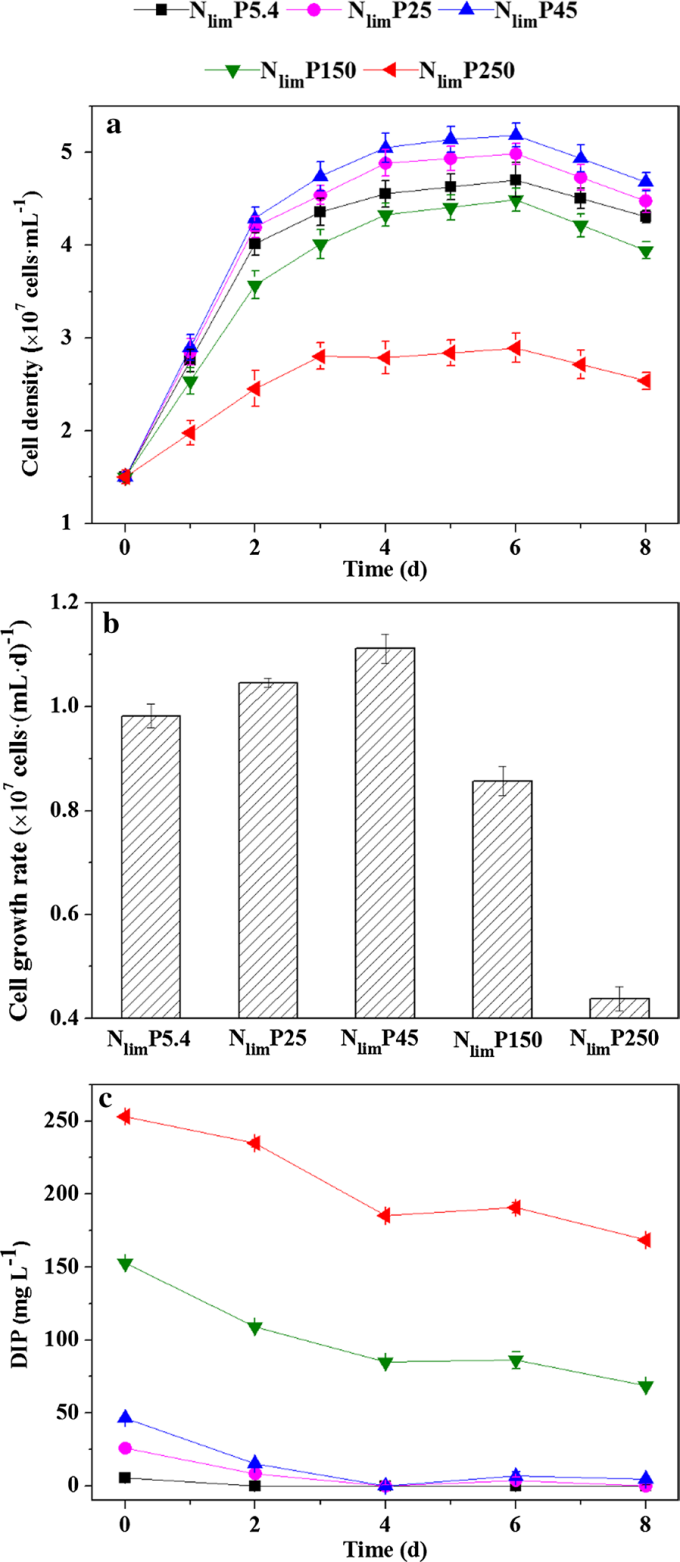
*C. regularis* growth profiles (**a**), the cell growth rate (**b**), and DIP consumptions (**c**) when P concentrations were 5.4 mg L^−1^ (N_lim_P5.4), 25 mg L^−1^ (N_lim_P25), 45 mg L^−1^ (N_lim_P45), 150 mg L^−1^ (N_lim_P150), and 250 mg L^−1^ (N_lim_P250). Error bars represent standard deviations, which were obtained based on triplicate measurements

Microalgal growth was evaluated via cell density [10], obtained with an optical microscope (BX53, Olympus, Japan) coupled with a hemocytometer. The results were determined based on the averages of at least three repetitions. The cell growth rate was calculated according to Eq. (1):

1 $${\text{Cell growth rate }}\left( {{\text{cell }}\left( {{\text{mL}}\;{\text{d}}} \right)^{ - 1} } \right) = (X_{ 2} - X_{ 1} )/\left( {t_{ 2} - t_{ 1} } \right)$$

where *X*_1_ and *X*_2_ (cell mL^−1^) represent the cell density at times *t*_1_ and *t*_2_, respectively; *t*_1_ and *t*_2_ represent the initial and final point within the linear portion of Fig. 1a.

Organics, phosphorus, and nitrogen consumptions were of particular concern, which were represented by the changing of chemical oxygen demand (COD), dissolved inorganic nitrogen (DIN), and dissolved inorganic phosphorus (DIP) in this work. These were determined via spectrophotometry using a water quality analyzer and the provided reagent kits (5B-3C V8, Lian Hua Technology, China) according to the manufacturer’s instructions. Prior to measurements, the samples were filtered with 0.45 µm cellulose acetate membranes to remove any suspended residues and biomass.

### Intracellular storage products: lipid, protein, and starch

The cells were collected on day 4 via centrifugation at 10,610×*g* for 10 min at 4 °C (TGL-16M, Cence, China), and were then freeze-dried (Pilot1-2LD, Boyikang, China) at − 80 °C. The achieved lyophilized powder was used for lipid extraction using the chloroform–methanol (2:1 v/v) reagent method [27]. Therefore, the lipid content could be calculated based on the gravimetric ratio of the extract and the powder and the lipid productivity was determined based on the following equation [28]:

2 $${\text{Lipid productivity }}\left( {{\text{mg (L}}\;{\text{d)}}^{ - 1} } \right) = (B_{2} \times C_{2} - B_{1} \times C_{1} )/(T_{2} - T_{1} )$$

where *B*_1_ and *B*_2_ represent the biomass (mg L^−1^) at the times *T*_1_ and *T*_2_, respectively; *C*_1_ and *C*_2_ represent the lipid content (%) at times *T*_1_ and *T*_2_, respectively. *T*_1_ and *T*_2_ represent the sampling time at initial and day 4.

To measure protein and starch, the cells were pretreated via ultrasound at 1500 W, − 4 °C for 30 min to break the cell-walls. Then, the solution was filtered with a 0.45 µm cellulose acetate membrane to remove fragments. The filtrate was ready for intracellular protein and starch evaluation. A folin-phenol assay kit (Dingguo Changsheng Biotechnology, Beijing, China) was employed for protein analysis [29]. For the hydrolysis of starch, 30% perchloric acid was added to the filtrate and stirred at 25 °C for 15 min, the extracts were analyzed with the anthrone method [30].

### Mitochondrial activity assay

The mitochondrial activity was determined with the 3-(4,5-dimethyl-2-thiazolyl)-2,5-diphenyl-2*H*-tetrazolium bromide (MTT) assay kit (Beyotime, Beijing, China) according to a previous publication [31]. Briefly, the cells were seeded to a 96-well plate (5 × 10^5^ cells mL^−1^) and were incubated with fresh air ventilated (5% CO_2_), at 37 °C. After 48 h, 10 µL MTT (5 mg mL^−1^) was added to the wells for another 4 h of incubation. The absorbance of the culture was measured at 570 nm using a microplate reader (RT-6000, Rayto, China).

### Morphology and ultrastructure of cells

Microalgal cell morphology was observed via optical microscopy (BX53, Olympus, Japan) and scanning electron microscopes (SEM) (XL-30 FE-SEM, FEI Co., USA). The ultrastructure of the cell was observed with a transmission electron microscope (TEM) equipped with an energy dispersive X-ray spectrometer (EDX) (JEM 1200EX, JEOL, Japan). Prior to SEM and TEM observation, the cells were pretreated by a series of multiple fixative and dehydrated procedures [32, 33]. Before TEM–EDX analysis, the dehydrated cells should be embedded in epoxy resin (Epon812, Shell Chemical, USA) for cutting (Microtome, Leica UCT, Germany) and ultrathin sections were further stained with uranyl acetate [34].

### ^31^P nuclear magnetic resonance spectroscopy (NMR)

The P-storage compounds in microalgal cells were extracted via the ice-cold HClO_4_ method [14, 35], and then 10% D_2_O was added to provide a field-frequency lock. The ^31^P NMR spectra were acquired at 400 MHz with a 5 mm probe using an NMR spectrometer (Vnmrs-300, Varian Co., USA). The acquisition time was 16 h at room temperature. MestReNova software 11 was used to analyze the peaks in the spectra, and the P compounds were identified by their chemical shifts based on the previously published spectra [35–37].

### X-ray photoelectron spectroscopy (XPS)

The binding energies of N1s and P2p were analyzed to investigate the character of the intracellular compounds via XPS (ESCALAB 250, Thermo, USA). The sample was the lyophilized powder of microalgal cells, which was obtained via the above-mentioned freeze-drying method. The beam source type was Al K Alpha, and it was operating at 250 W, a voltage of 15 kV, a current of 15 mA, an energy of 1486.71 eV; the sampling spot size was 500 µm in diameter and an electron takeoff angle of 50° was used. The binding energy was calibrated with reference to C1s at 284.8 eV in the spectra analysis with XPS Peak 4.1 software [38].

### Statistical analysis

P concentration effects were investigated based on triplicate cultivations, and then the statistically significances were analyzed using one-way ANOVA method. The mean values were compared using a least significant difference (LSD) test. Differences were significant when *P* < 0.05. All statistical analyses were performed using the SPSS software package (IBM SPSS statistic 20.0).

## Results and discussion

### Effect of P concentrations on *C. regularis* growth

The cell density of *C. regularis* was ~ 4.70 × 10^7^ cell mL^−1^ at the stationary stage when P supply was regular, N_lim_P5.4 (refers to the classic BG11), see Fig. 1a. The cell density was about 76% of that in NP5.4 [18], indicating that nitrogen limitation inhibited cell growth. Microalgal growth was stimulated when P was slightly excessive, i.e., the cell density increased by 10.2% for N_lim_P45 (*P* < 0.05). A considerable number of previous studies reported that such a stimulation was caused by storage of excess P, under either autotrophic or heterotrophic cultivations [4, 14, 39, 40], where nitrogen was either limited or unlimited [41]. Excessive P induced improvements on supplying phospholipid, genetic materials, and energy for cell division [42, 43]. However, further increasing of the P supply could result in microalgal growth inhibition. The cell density decreased for N_lim_P150 and decreased as much as 38.8% for N_lim_P250, compared to the control (N_lim_P5.4) (*P* < 0.05). In addition, the growth rate in Fig. 1b intuitively shows that the effects of P depended on its concentration (*P* < 0.05). These results indicate that *C. regularis* growth was promoted when P was at an appropriate level; however, a large excess of P had a negative impact on the cells. This is a typical manifestation of hormesis [44, 45].

Glucose, nitrogen, and phosphorus uptake profiles were represented by COD, DIN, and DIP changing versus time, respectively, see Additional file 1: Fig. S1 and Fig. 1c. The organic and nutrient consumptions were consistent with the growth profiles: stimulated by low P concentrations (P ≤ 4.49 × 10^−7^ mg cell mL^−1^) and slowing down at high P levels (P ≥ 14.97 × 10^−7^ mg cell mL^−1^). It is worth mentioning that P (PO_4_^3−^–P) was completely consumed within 4 days when P was in slight excess (less than 45 mg L^−1^); in contrast, a large amount of P (PO_4_^3−^–P) remained for both N_lim_P150 and N_lim_P250 (Fig. 1c). These results implied that the capability of P storage reached a threshold, above which, P may cause microalgal cell damage.

### Effects of P concentration on lipid productivity

**Fig. 2:**
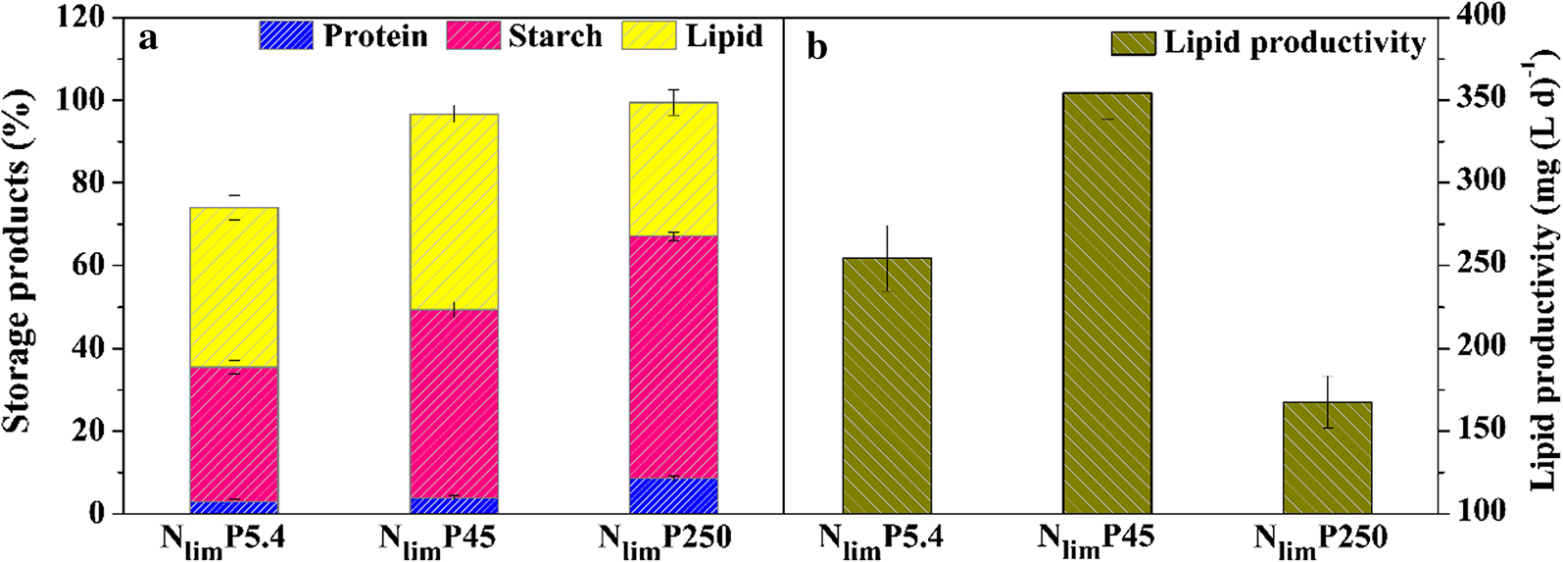
Effects of P concentrations on *C. regularis* intracellular components. Lipid, protein, and starch content (**a**), and lipid productivity (**b**) on day 4. N_lim_P5.4, N_lim_P25, N_lim_P45, N_lim_P150, and N_lim_P250 represent the protocols using the modified BG11 media with P concentrations of 5.4, 45, 150, and 250 mg L^−1^, respectively. Error bars were obtained based on triplicate measurements

It seemed that P supply affected the carbon flow of the microalgal glucose metabolism. The lipid content enhanced by 22.9% for slightly excessive P (N_lim_P45) (*P* < 0.05). However, lipid synthesis was significantly suppressed when *C. regularis* cells were inhibited by a large excess of P (N_lim_P250), whereas the lipid content decreased by 15.9% in comparison to the control (N_lim_P5.4) (*P* < 0.05). For the cell density (Fig. 1a), the highest lipid productivity of 354.38 mg (L d)^−1^ was achieved for N_lim_P45, which increased by 39.3% in comparison to the control (*P* < 0.05). However, the lipid productivity of *C. regularis* for N_lim_P250 was only 47.3% of N_lim_P45 and decreased by 34.2% in comparison to control (*P* < 0.05). Accordingly, the intracellular protein and starch contents both positively correlated with P supply, as shown in Fig. 2a.

It has previously been reported that P enhances both microalgal lipid synthesis and production. P-replete favored the enzymes of the up-regulation of the lipid synthesis pathway [40] and could enhance the biomass production by activating phosphofructokinase and pyruvate kinase related glycolysis [46]. Here, for the first time, the toxicity of a large excess of P was addressed in microalgae. Excessive P dosage does not always increase the lipid production of microalgae under nitrogen limitation.

### Hormesis mechanism

#### Effect of P concentration on cell viability

**Fig. 3:**
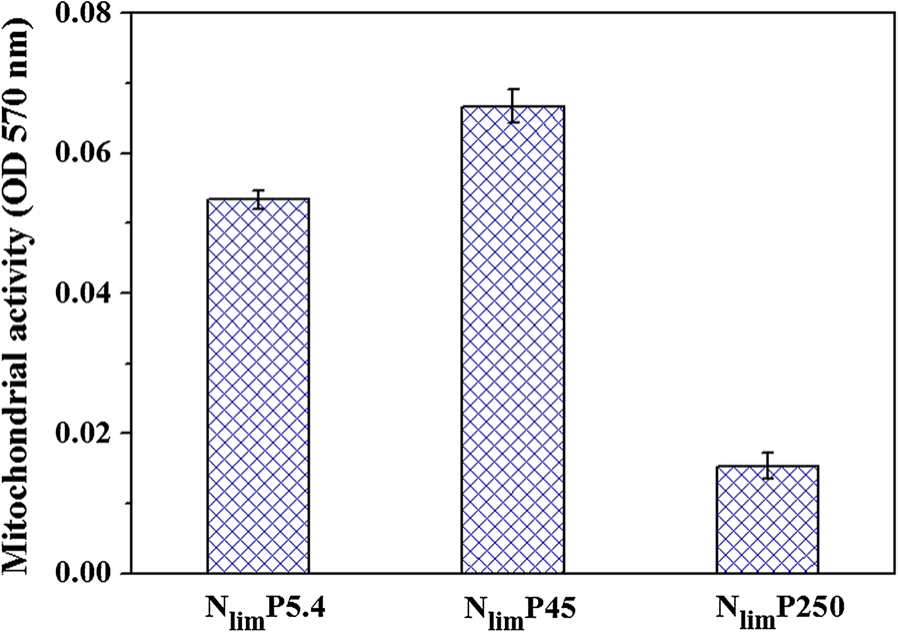
Effect of P concentrations on mitochondrial activity of *C. regularis* under nitrogen limitation, with P 5.4, 45, and 250 mg L^−1^ (N_lim_P5.4, N_lim_P45, and N_lim_P250, respectively). Error bars were obtained based on triplicate measurements

Mitochondrial activity was used to study the microbial viability [31, 47], since more than 95% energy is generated in mitochondria, which are the “powerhouses” of the cell [48]. In this work, the trend of mitochondrial activity again supported P hormesis deduction, as shown in Fig. 3. Mitochondrial activity was stimulated and consequently increased by 25.0% (*P* < 0.05), when P was only slightly excessive (N_lim_P45). However, it decreased as much as 71.3% for N_lim_P250 (*P* < 0.05), indicating dysfunction of mitochondria due to the toxicity of the large excess of P.

#### Cell morphology and ultrastructure

**Fig. 4:**
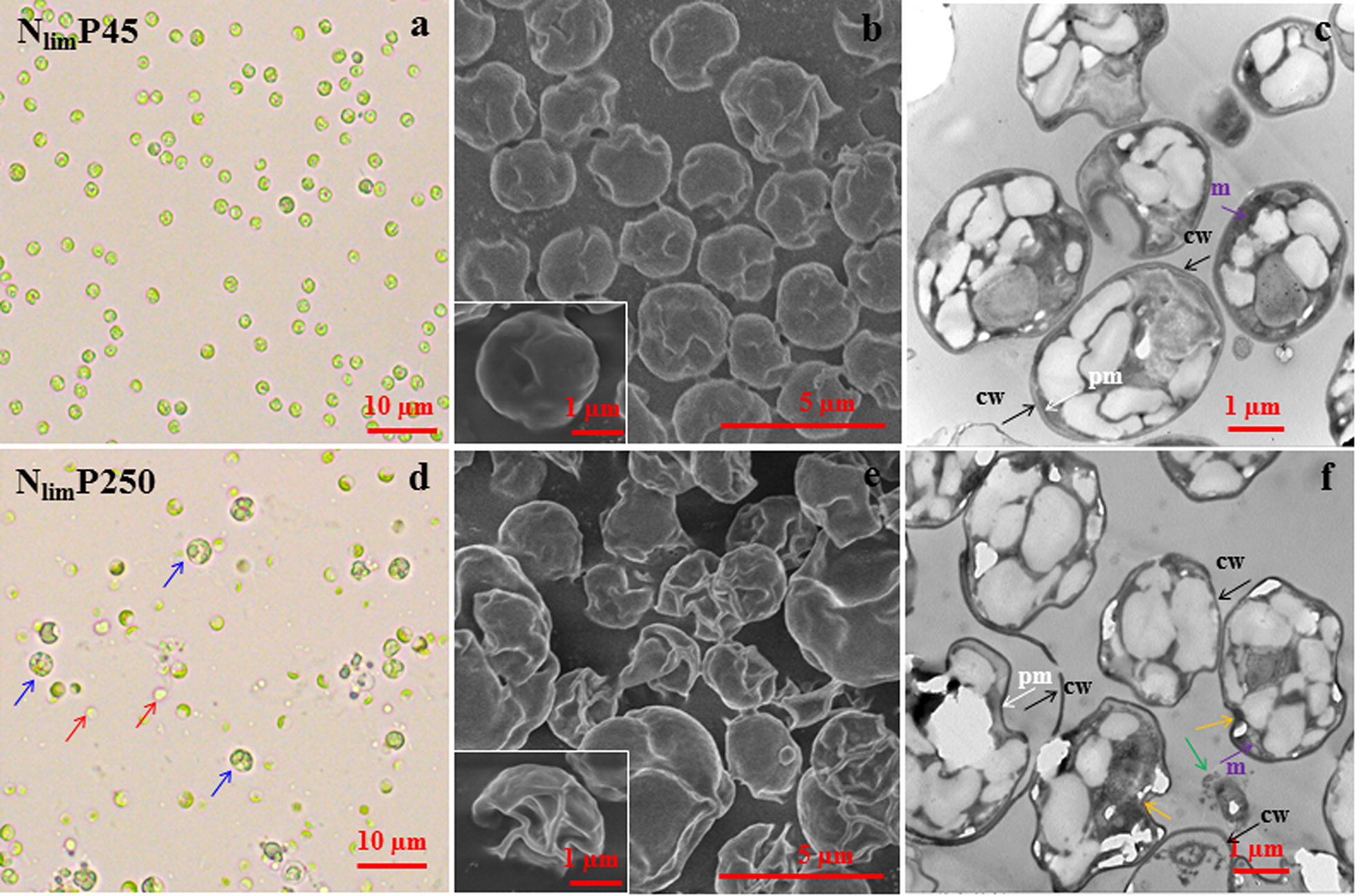
Morphology of surface and inner *C. regularis* cells cultivated under two conditions (nitrogen limitation and P 45 mg L^−1^, N_lim_P45, **a**–**c**; nitrogen limitation and P 250 mg L^−1^, N_lim_P250, **d**–**f**) with beneficial and inhibition effects for growth. The subfigures in **b** and **e** show representative enlarged views of cells. The red, blue, orange, and green arrows indicate light colored cells, large sized cells, fold structure, and lysed cells devoid of recognizable contents, respectively. *cw* cell wall, *pm* plasma membrane, *m* mitochondria

Cell morphology and ultrastructure further corroborated the hormesis mechanism of P. *C. regularis* cells of N_lim_P45 had a round shape, integrated structure, and intact organelles (Fig. 4a–c). All of these characteristics were identical to control cells. In contrast, the cells became folded for N_lim_P250, and several cells enlarged, reaching up to two- to three-fold of the size of the control (Fig. 4d, e). TEM images further showed that the cell walls thinned and even detached. Such damage of the plasma membrane and the following plasmolysis caused the described cell surface folding [49]. In addition, several organelles, such as mitochondria, were disordered when P was in large excess (Fig. 4f). These signs indicate targeted P poisoning of microalgal cells.

### Intracellular P distribution and the role of polyphosphates

**Fig. 5:**
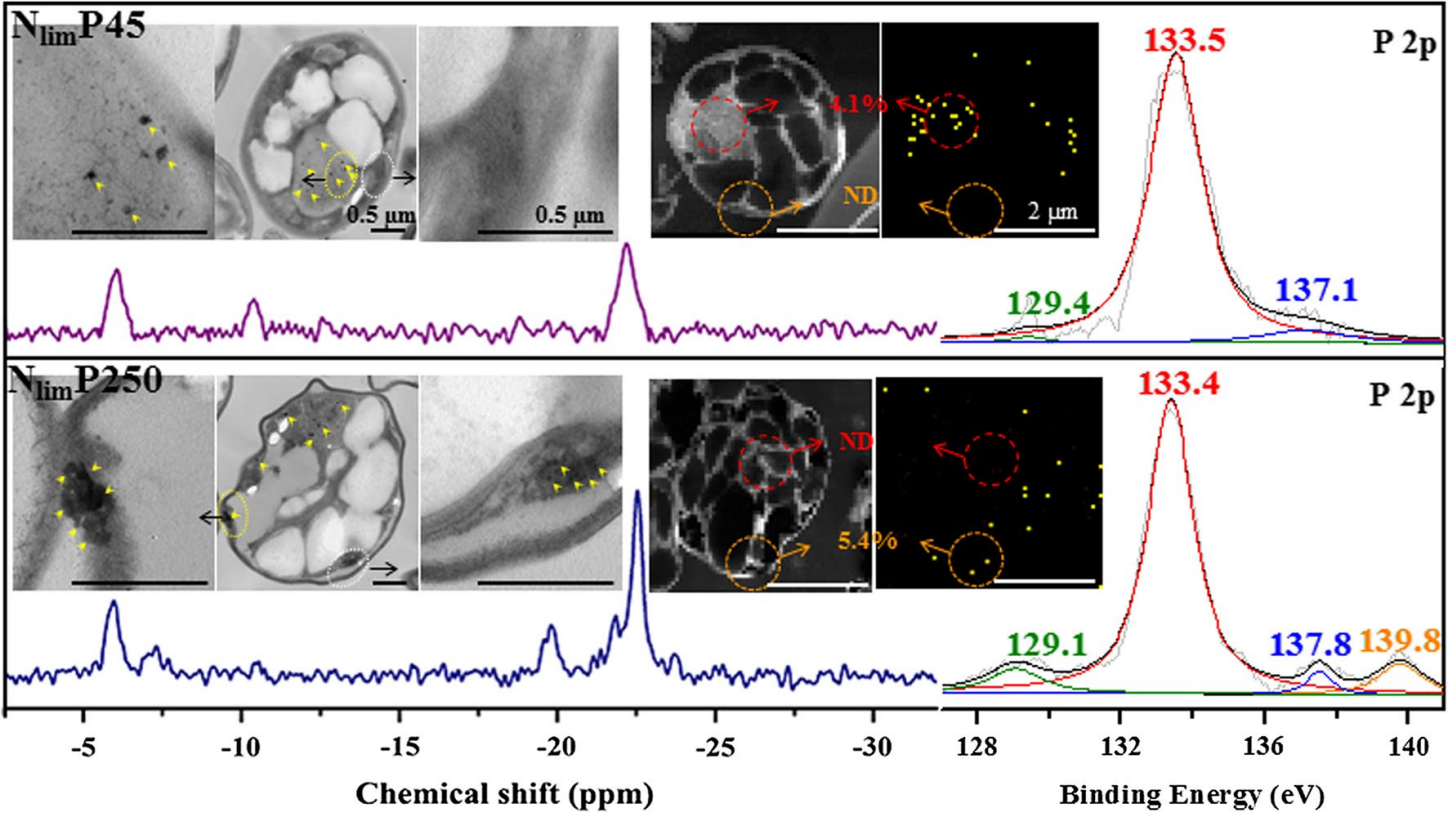
^31^P NMR spectra and P 2p XPS spectra in *C. regularis* cells, with magnified TEM and P elemental mapping images inserted. The yellow arrows indicate poly-P granules. The red circle shows the inner cell area, the orange circle shows the cell wall area. The data from the red and orange circles indicates the weight percentage of P in the circle area, ND means not detected

P distributions in *C. regularis* cells were different between stimulated (N_lim_P45) and inhibited (N_lim_P250) cell growth. P was mainly found in the intracellular region for N_lim_P45 (4.13% in weight), while it was located at the cell periphery for N_lim_P250 (5.4% in weight), see Fig. 5. P in *C. regularis* formed poly-Ps (small black spots in TEM) in the cells for N_lim_P45, which were at the resonances in − 5 to − 7 and − 17 to − 22 ppm [35, 36] in the ^31^P NMR spectra (Fig. 5). The poly-Ps also convinced by the peaks at 129.4 eV and 137.1 eV [50] in P 2p XPS spectra (Fig. 5). Poly-P served as an energy pool to stimulate cell division and metabolism [37].

However, the forms of poly-P became different when the excess poly-P was stored for *C. regularis* (N_lim_P250). The poly-Ps located near the cell periphery, which likely damaged both plasma membrane and cell wall (Fig. 5). In addition, mitochondria were disordered, as the peaks (− 11.5 ppm) of ATP and NADH [35] almost disappeared in ^31^P NMR spectra for N_lim_P250 (Fig. 5). This indicated that excess poly-Ps damaged the energy production process, which was consistent with the results on growth inhibition and mitochondrial activity decrement. Consequently, the excess poly-P accumulation disintegrated the thylakoid membranes and resulted the cell lysis and cell death at the end [51]. Moreover, excess poly-P was able to form dinucleoside polyphosphates with similar structure to ATP and other essential mononucleotides; therefore, it was also toxic for cells and caused DNA damage in the intra-S phase [52].

**Fig. 6:**
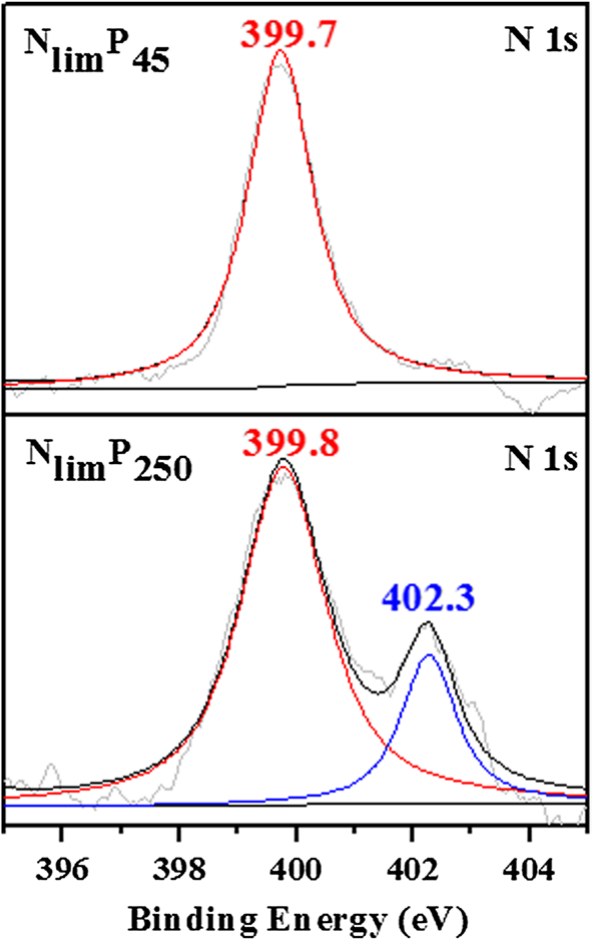
N 1s XPS spectra of *C. regularis* cells. The microalgal cells were freeze-dried to obtain lyophilized powder for XPS analysis

Excess poly-P could bind to intracellular components and inhibit cell viability [18]. There were several new peaks at − 7.7 ppm and − 19.8 ppm in the ^31^P NMR spectra, which indicated that the new form of P appeared for N_lim_P250 (Fig. 5). This was also confirmed by the peak at higher binding energy of 139.8 eV in the P 2p XPS spectra (Fig. 5), which represented the binding compounds of poly-P to intracellular components [18]. Furthermore, a new peak of protonated amide-N in protein at 402.3 eV [53, 54] appeared for N_lim_P250 (Fig. 6). It seems that the excess poly-P bonded to the proteins and resulted protonated amide-N, which affected the binding of the protein–ligand [55]. Thus, a major constraint in protein breakage and large cytoplasmic crevices appeared, followed by interfered interactions of protein subunits [55]. The changes on channel and transporter proteins induced the across membrane process of ions and disordered small molecules [56]. In this way, membrane permeability was damaged and membrane proteins were disrupted [57].

## Conclusions

P presented a hormesis effects for microalgal cultivation. Slightly excessive P levels (≤ 45 mg L^−1^) stimulated *C. regularis* enhancement of growth (10.2%), lipid accumulation (22.9%), and mitochondrial activity (25.0%) via poly-P storage energy. The total lipid productivity increased by 39.3%. In contrast, large excess of P (P ≥ 150 mg/L) poisoned *C. regularis* cells, which showed enlarged size, plasmolysis, deformation of cell walls, and disorganization of organelles. Both cell density and mitochondrial activity decreased by 38.8% and 71.3%, respectively, followed by a decrease of the final lipid productivity of 34.2%. The poisoning mechanisms are related to intracellular amide-N in protein protonation and the damage of the plasma membrane.

## Additional file

**Additional file 1: Fig. S1.** The COD and DIN consumptions during *C. regularis* growth with different P concentrations. Error bars represent standard deviation values, which were obtained based on triplicate measurements.

## Data Availability

Not applicable.

## Abbreviations

P: phosphorus
NADPH: reduced nicotinamide adenine dinucleotide phosphate
NADP: nicotinamide adenine dinucleotide phosphate
ATP: adenosine triphosphate
ADP: adenosine diphosphate
poly-P: polyphosphate
DNA: deoxyribonucleic acid
RNA: ribonucleic acid
*C. regularis*: *Chlorella regularis*
N_lim_: nitrogen limitation
COD: chemical oxygen demand
DIN: dissolved inorganic nitrogen
DIP: dissolved inorganic phosphorus
MTT: 3-(4,5-dimethyl-2-thiazolyl)-2,5-diphenyl-2*H*-tetrazolium bromide
SEM: scanning electron microscopes
TEM: transmission electron microscope
EDX: energy dispersive X-ray spectrometer
NMR: nuclear magnetic resonance
XPS: X-ray photoelectron spectroscopy
