# Supplementary material for: Hormesis effects of phosphorus on the viability of Chlorella regularis cells under nitrogen limitation
Source: Biotechnol Biofuels. 2019 May 13;12:121. doi: 10.1186/s13068-019-1458-z (PMC6513516; doi:10.1186/s13068-019-1458-z)
Supplement: Supplementary file 1 — Additional file 1: Fig. S1. The COD and DIN consumptions during C. regularis growth with different P concentrations. Error bars represent standard deviation values, which were obtained based on triplicate measurements. [file 13068_2019_1458_MOESM1_ESM.docx]

**Additional file 1**





**Fig. S1.** The COD and DIN consumptions during C. regularis growth with different P concentrations. Error bars represent standard deviation values, which were obtained based on triplicate measurements.
